# Strength Optimisation of Hybrid Bolted/Bonded Composite Joints Based on Finite Element Analysis

**DOI:** 10.3390/ma17133354

**Published:** 2024-07-06

**Authors:** Raphael Blier, Leila Monajati, Masoud Mehrabian, Rachid Boukhili

**Affiliations:** 1 Department of Mechanical Engineering, Royal Military College of Canada, Kingston, ON K7K 7B4, Canada; raphblier@gmail.com; 2 Department of Mechanical Engineering, Polytechnique Montréal, Montreal, QC H3T 1J4, Canada; leila.monajati@polymtl.ca (L.M.); masoud.mehrabian86@gmail.com (M.M.)

**Keywords:** finite element analysis, composite materials, hybrid bolted/bonded joints, design optimisation, load-sharing phenomenon

## Abstract

A finite element analysis (FEA) was conducted to examine the behaviour of single-lap quasi-isotropic (QI) and cross-ply (CP) hybrid bolted/bonded (HBB) configurations subjected to tensile shear loading. Several critical design factors influencing the composite joint strength, failure conditions, and load-sharing mechanisms that would optimise the joining performance were assessed. The study of the stress concentration around the holes and along the adhesive layer highlights the fact that the HBB joints benefit from significantly lower stresses compared to only bolted joints, especially for CP configurations. The simulation results confirmed the redundancy of the middle bolt in a three-bolt HBB joint. The stiffness and plastic behaviour of the adhesive were found to be important factors that define the transition of the behaviour of the joint from a bolted type, where load sharing is predominant, to a bonded joint. The load-sharing potential, known as an indicator of the joint’s performance, is improved by reducing the overlap length, using a low-stiffness, high-plasticity adhesive, and using thicker laminates in the QI layup configuration. Enhancing both the ratio of the edge distance to the hole diameter and washer size proves advantageous in reducing stresses within the adhesive layer, thereby improving the joint strength.

## 1. Introduction

The mechanisms of damage initiation and bolt–adhesive interaction are important features characterising the performance of hybrid-bolted-bonded (HBB) joints. The bolt–adhesive interaction can take the form of load sharing or a reduction in the peel stresses at the overlap ends. According to the preliminary results, HBB joints offer superior performance compared to bolted joints but also to bonded joints due to the crack-stopping feature of the bolts [1,2]. This indicates that the bolts must have a definite impact on the failure of the adhesive layer. Bodjona and Lessard [3] reported that less than ten percent of the applied load was found to be transferred by the bolt in a single-lap (SL) HBB joint when the adhesive was not fully plasticized. Moreover, controlling the adhesive thickness and minimising the bolt–hole clearance are efficient methods to limit the maximum plastic strain. In other work, Bodjona et al. [4] observed that, considering the initial failure of a low-compliance adhesive, no advantage was observed from adding a fastener to a bonded joint, while it significantly postponed the initial failure of a high-compliance adhesive.

FEA as a powerful tool for applied numerical modelling of structures has been extensively used by researchers [5,6,7]. Using finite element analysis (FEA), Kelly [8] investigated the effects of load sharing in SL HBB joints, considering the effects of increasing the adherend and adhesive thickness and decreasing the overlap length, bolt pitch distance, and adhesive modulus on higher load transfer. Li et al. [9] investigated various parameters in HBB joints and concluded that selecting a high-strength, low-modulus adhesive is preferable for improving the bonding strength. It was also mentioned that the HBB acts like a bonded joint before adhesive failure, while it performs like a bonded joint after adhesive failure. In order to increase the load sharing before adhesive failure, Raju [10] designed an interference-fit HBB joint instead of a conventional neat-fit or clearance-fit bolt. The model achieved ten percent higher load sharing considering equivalent load levels. Romanov et al. [11] showed the importance of joint overlap length on joint strength rather than bolt positioning. It was concluded that shorter overlap lengths and smaller bolt-edge distances led to higher load sharing. Several researchers have studied the mechanical characteristics of HBB joints, considering different bolt configurations [12,13,14,15] resulting in various conclusions as to the factors that enhance their performance [16,17,18].

Researchers have already established that load sharing, a characteristic of the load distribution between the adhesive and fasteners, is a crucial factor influencing the performance of HBB joints. In the current research, the geometric parameters and adhesive properties are investigated to see whether they allow proper load sharing before joint failure. Additionally, the influence of geometric parameters on the bolts’ contribution to the reduction in peel stresses in the adhesive is analysed. Digital image correlation (DIC), as one of the powerful tools for measuring the strain field and recognising the onset of failure around the fastener [19], has already been used to compare the experiments with the FEA results. Through the use of the 3D-DIC technique, the experimental results using the current model were validated with the FEA results determined in the previous article by the same authors [20]. Using the same simulation modelling, several optimisation parameters are investigated to study the influence of the bolt in HBB joints, including the influence of the middle bolt, the load-sharing potential with changing overlap lengths, adhesive properties, and laminate thickness, the effect of the e/d ratio, and the washer size.

## 2. Materials and Methods

### 2.1. Model Description

**Figure 1:**
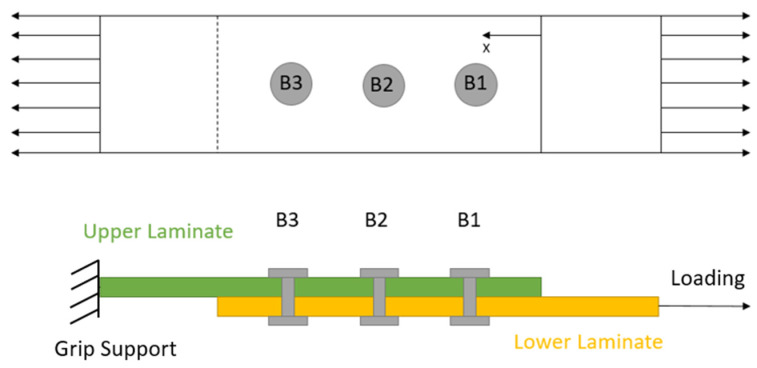
Joint model.

The joint model considered in this study is shown in Figure 1, considering B3 as the nearest hole from the grip support and B1 as the furthest one. It should be noted that the results for the adhesive, such as the shear or peel stress distributions, are taken in the middle of the adhesive (where the cohesive interaction is defined). For the following research, the failure of the joint is defined as the onset of fibre or adhesive damage. This was chosen as it reduced the length of the simulation while still being a good indicator of the relative strength of the different joint configurations.

In order to study laminates, three types of joint configurations, including only bolted (OB), only bounded, and hybrid bolted-bonded (HBB), are considered. Two critical layups, including cross-ply (CP) and quasi-isotropic (QI), considering 12 plies, are described in Table 1 according to the previous work done by the same authors [20]. The abbreviation CP12 signifies a 12-ply cross-ply layup, as an example.

### 2.2. Simulation Procedure

The FEA simulations were run using Abaqus/CAE 2019 (4RealSim, Jsselstein, The Netherlands) An explicit non-linear model was used for this analysis, as large deformations occur at failure, multiple contacts are defined, and damage evolution is included in the analysis. A full description of the procedure followed for modelling the specimen, considering its adhesive and composite material properties based on the classical plate theory, damage modelling, and mass scaling, was well-described in the previous article by the same authors [20]. In the research mentioned, in order to ensure the FE results, they were compared with the experimental procedures using the 3D digital image correlation method. The washers and bolts were modelled considering the temperature gradients for each joint configuration, and the special considerations regarding contact definitions were completely explained. It should be noted that a specific technique followed by Gordon et al. [21] was followed to model plain weave fabric layers by substituting each fabric layer with four symmetric unidirectional plies.

**Figure 2:**
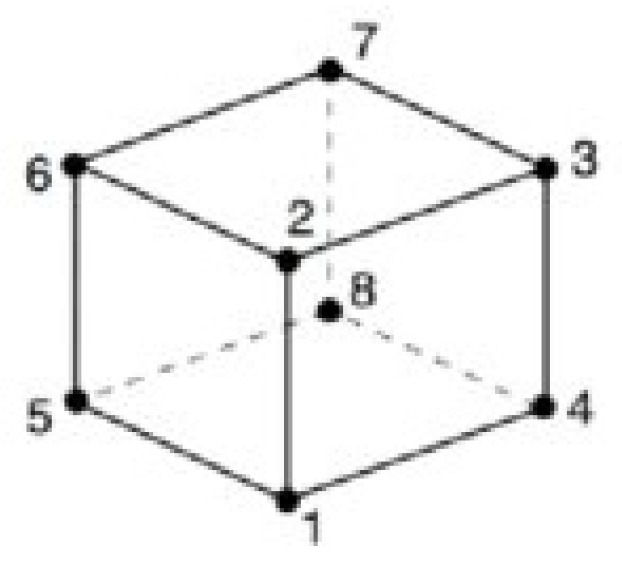
First-Order Solid Hexahedral Reduced Integration (C3D8R) Element.

In order to capture the high stress gradients around the holes, first-order solid hexahedral reduced integration (C3D8R) elements were used for the entire model. These elements have 1 integration point and 8 nodes, as shown in Figure 2. Each node is capable of only a translation degree of freedom (DOF), giving a total of 24 degrees of freedom to these elements.

In order to reduce the computational cost and avoid shear locking, a common problem with fully integrated solid elements, the reduced integration scheme was used. However, the reduced integration elements can suffer from hourglassing due to the lower number of integration points. Thus, an enhanced hourglass control algorithm was selected to artificially increase stiffness and mitigate the risk of hourglassing in the element.

In the case of HBB joints, the mesh convergence study was monitored for the initiation of cohesive damage as a function of the stress state at the adhesive cohesive interaction interface. Due to the presence of adhesive in HBB joints, high stresses develop at the overlap ends. Therefore, the mesh had to be refined at this location to capture this high stress gradient. As every layup has the same geometry, the mesh convergence study was only performed for the CP12 layup, and the converged mesh was used for all models. The mesh convergence study for HBB joints is shown in Table 2.

**Figure 3:**
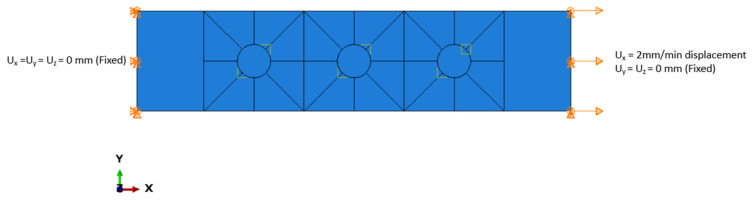
OB and HBB joints model boundary conditions (shown for QI layup).

For the joint configurations (OB or HBB), the end faces of the upper laminate on the grip side have a prescribed displacement of 0 in all directions, while the end faces of the lower laminate on the grip side have a prescribed displacement of 0 in the y and z directions with a prescribed displacement in the x direction at a rate of 2 mm/min as shown in Figure 3. The CP layups have a symmetry BC with respect to the x-z plane, and only half of the joint is modelled. The geometries were partitioned around the holes to help produce a high mesh quality. Additionally, in OB and HBB joints, the bolts were modelled as one component, where the head and the nut were assumed to have identical geometries.

**Figure 4:**
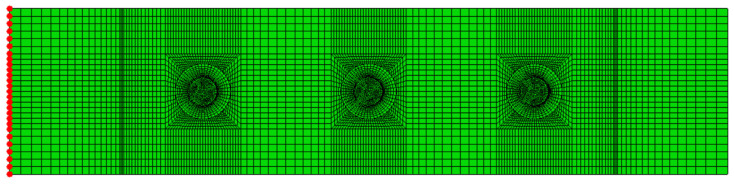
Nodes (in red) at which reaction forces are extracted (shown for QI layup).

The reaction force used to evaluate the applied load is computed by summing the axial reaction forces taken at the nodes on the constrained grip side, as shown in Figure 4. In the case of CP layups, this sum is multiplied by a factor of 2, as only half the joint is modelled.

## 3. Results

### 3.1. Stress Analysis

#### 3.1.1. Stress Concentration in the Laminate in HBB and 3OB Joints

**Figure 5:**
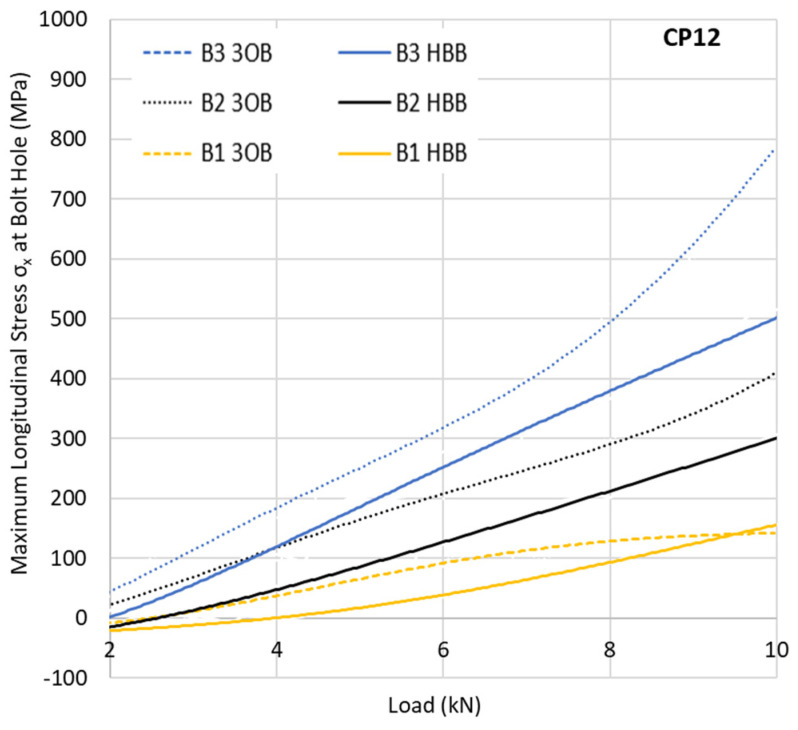
Maximum longitudinal stress σ_x_ at different holes in CP layups.

**Figure 6:**
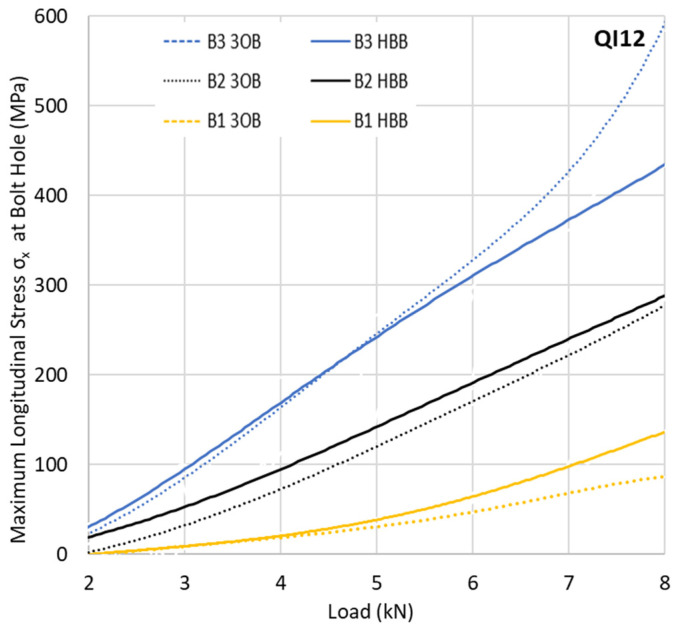
Maximum longitudinal stress σx at different holes in QI layups.

The critical state of the stress at the hole that generates failure consists of the combination of σ_x_ and a shear component S_xy_. A variation in the peak stress at all hole locations is of great interest to see how the joining method influences the development of those high stresses. The predicted variation in the normal stress with the imposed loading at the critical point of each hole of the top ply is depicted in Figure 5 and Figure 6 for the 3OB and HBB joints consisting of CP and QI layups. Due to the bolt preload, negligible compressive stress (around 10 MPa) is observed at very low load levels as the laminate is compressed, creating a compression zone in the bending.

**Figure 7:**
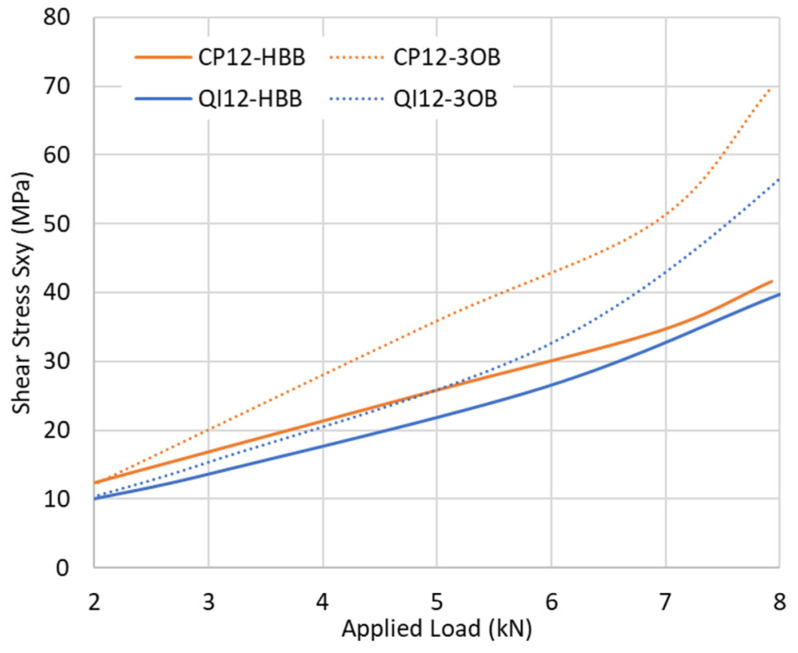
Maximum shear stress S_xy_ in 0° plies at hole B3 for the CP12 and QI12 joints.

For the QI layup, a slight reduction in the maximum longitudinal stress is observed for the HBB joints. However, this difference is much less apparent than in the case of the CP layup. The difference in behaviour between the CP and QI layups can be explained by the fact that CP layups are more notch sensitive, a phenomenon reduced by the adhesive layer. This notch sensitivity relates not only to the longitudinal stresses σ_x_ but also to the shear stresses S_xy_. The CP layups suffer matrix damage at a lower load level compared to the QI layups due to the development of higher shear stresses, reducing the local stiffness as shown in Figure 7 for the critical point (at hole B3). These data indicate a net gain in the reduction in the shear stress generated by the adhesive, independent of the layup. This gain becomes even more substantial with increased loading. However, due to the notch sensitivity of the CP layups, the effect is more significant, reducing the matrix damage significantly. For the CP layups, the reduction in the maximum shear stress around the holes due to hybridisation is almost double compared to a QI layup, showing the significant gains that can be achieved, especially for CP HBB joints.

#### 3.1.2. State of Stress Comparison in the Adhesive in the HBB and Bonded Joints

**Figure 8:**
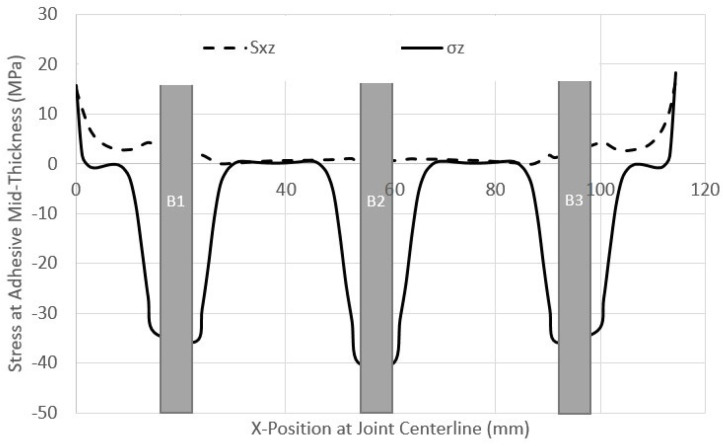
Stress distribution along the centreline of the adhesive layer for the CP12-HBB joint at 8.5 kN.

The overlap ends are the regions of interest, as high stresses, in the form of peel and shear, develop in the adhesive at these locations, as shown in Figure 8.

**Figure 9:**
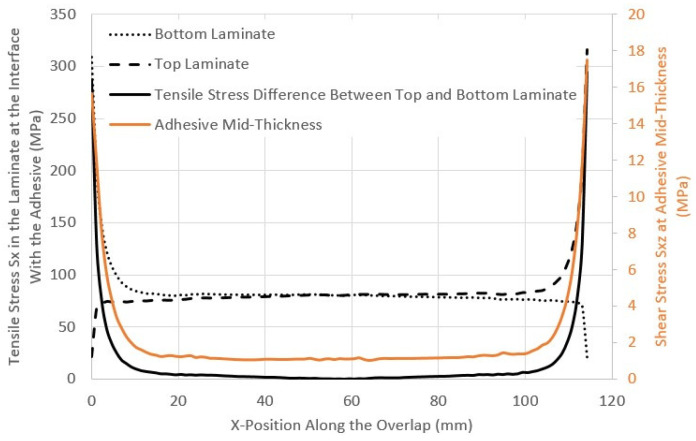
Stress distribution in the laminate and the adhesive in CP12-HBB joints at 8 kN.

The difference in the longitudinal stress in the laminate at the adhesive interface at the overlap end is depicted in Figure 9. The large difference will result in the shearing of the adhesive. The increase in the difference in longitudinal stress in the laminates matches the increase in shear stress in the adhesive layer. The difference in the longitudinal stress between the top and bottom laminates is attributed to the fact that at the beginning of the overlap, no load was transferred in one laminate, while all the load was transferred by the other one. The difference in longitudinal tensile stress is also increased by secondary bending.

**Figure 10:**
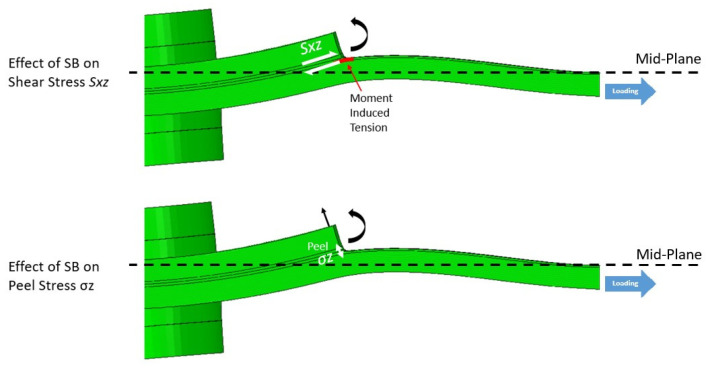
Effect of the secondary bending on the HBB joint, in the case of the CP12-HBB Joint at 8 kN (Deformation Scale 50×).

As shown in Figure 10, secondary bending increases the difference in the tensile stress between the two laminates by creating an additional tensile stress component due to the moment induced by the eccentricity in the load path. Additionally, the secondary bending phenomenon causes high peel stresses as the laminates are forced away from each other but maintained together by the adhesive. This pulling-apart action by the laminate generates tensile stress in the adhesive layer. The secondary bending also affects the shear stresses S_xz_ at the overlap end due to the joint’s rotation. The increase in tensile stresses due to secondary bending generates a larger difference in the longitudinal stresses between the top and bottom laminates, resulting in higher shear stresses.

**Figure 11:**
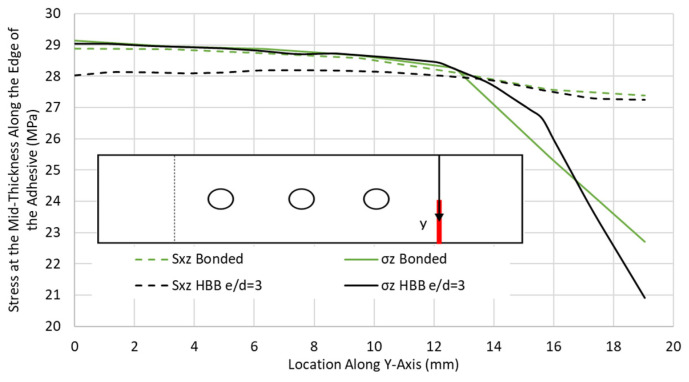
Peel σ_z_ and shear stress S_xz_ distribution along the adhesive edge in the QI12 HBB and bonded joints at 12.5 kN.

The case of a QI-HBB joint at 12.5 kN (before failure) was chosen to illustrate the variation in the critical stresses along the edge of the adhesive (*y*-axis), as shown in Figure 11. Similar results obtained for only bonded joints were added for comparison. It is interesting to note that the highest peel stresses were located along the joint centreline. These preliminary results indicate that the bolts are redundant, in the sense that failure is dictated by the stress levels at the edge of the adhesive layer, and these are not impacted by the presence of the bolt in the current HBB joint configuration.

From this investigation, the key benefits of hybridisation for a bolted joint have been highlighted by the reduction in the shear stresses as well as the reduction in the tensile stress at the holes provided by the adhesive layer. However, the holes still generate considerable stress concentrations in the HBB joint. Thus, the HBB joint design must optimise the role of the bolts such that they contribute to a reduction in the stresses in the adhesive layer to justify the presence of such discontinuity in the laminates.

Thus, the next section focuses on the potential parameters that could permit stress reduction in the adhesive layer by the bolts. Several parameters are investigated to study the influence of the middle bolt in three-bolt HBB joints: the potential load sharing while changing the overlap lengths, adhesive properties, and laminate thickness; and the effects of the e/d ratio and washer size on the stresses in the adhesive layer.

### 3.2. Investigation of Design Parameters to Optimise HBB Joints

#### 3.2.1. Role of the Middle Bolt in Three-Bolt HBB Joints

Improvements in the HBB joint design may take the form of an increase in the joint load-carrying capability but also a joint weight reduction for the same load-carrying capability. The latter is investigated first, as Gamdani et al. [2] indicated a potential redundancy of the middle bolt in three-bolt HBB joints. For that reason, models were developed with the same overlap length; however, one had three bolts and the other had two bolts. Simulations were run for CP and QI layups, and it was found that the removal of the middle bolt did not influence the failure load of the joint, as shown in Table 3.

**Figure 12:**
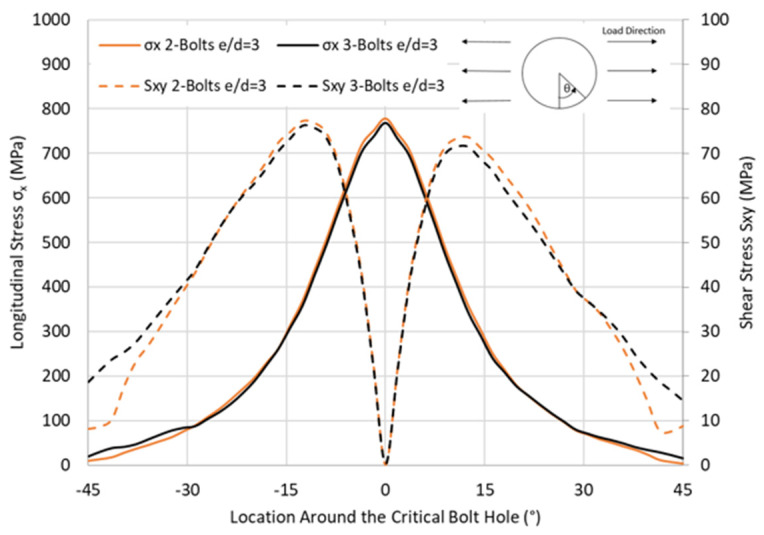
Effect of the middle bolt in the tensile σ_x_ and shear stress S_xy_ distribution around the critical hole in CP12-HBB joints at 14 kN.

It was shown previously that, at B3, located on the grip side, the longitudinal tensile stresses were much higher than at the hole on the free side, making this location the focus for the stress level comparison. In order to understand the reason for the similar joint strength upon the removal of the middle bolt, stresses around the critical hole were monitored for the HBB joint using CP laminate, as shown in Figure 12. It is found that the presence of the middle bolt does not alleviate the stresses at the critical hole. This is due to the load being transferred uniquely by the adhesive, a common phenomenon when using stiff adhesive in HBB joints, which means that the stress levels are independent of the presence of the middle bolts.

**Figure 13:**
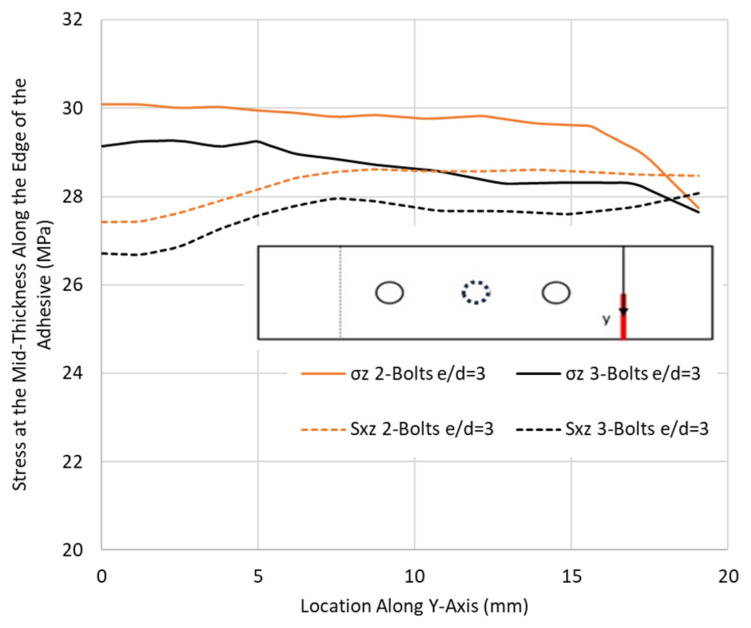
Effect of the middle bolt on the peel σ_z_ and shear stress S_xz_ at the overlap ends along the adhesive edge in the CP12-HBB joints at 14 kN.

Similarly, the stresses were monitored in the adhesive at the overlap end. As was the case for the stresses at the critical hole, there is no significant difference between the two configurations. The stress distribution shown in Figure 13, for the CP case taken as a basis for this comparison, highlights that, although slight discrepancies are observed, the removal of the middle bolt did not affect the stresses in the adhesive layer. As such, for an overlap length requiring more than one bolt to maintain e/d = 3, it is recommended that only two bolts be used. The bolts between the outer bolts are considered redundant and will only add weight to the joint.

#### 3.2.2. Load Sharing

**Figure 14:**
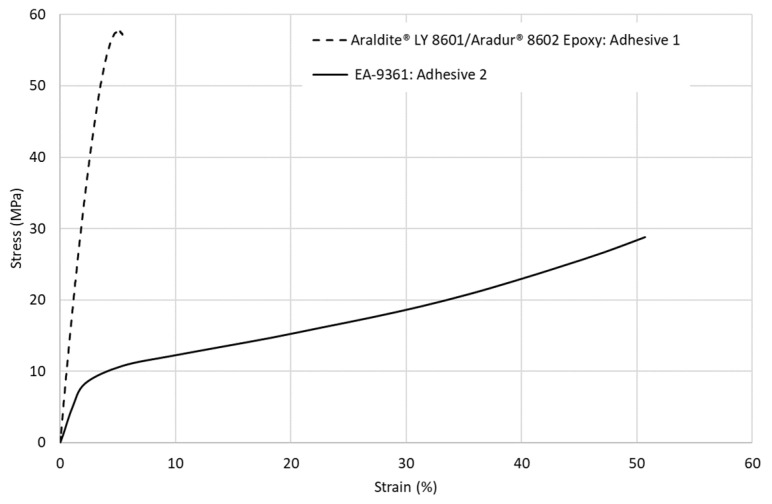
Tensile stress–strain curves of adhesives 1 and 2.

To investigate the load-sharing phenomenon, two adhesives are selected. Adhesive 1 is the material used so far for the investigation, Araldite^®^ LY 8601/Aradur^®^ 8602 Epoxy provided from Huntsman corporation, Conroe, TX, USA, a stiff brittle adhesive used in the experimental tests. Adhesive 2 is the EA 9361 Epoxy provided from Henkel-Hysol corporation, Mississauga, ON, Canada, a high-plastic deformation and low-stiffness adhesive commonly used in load-sharing studies. The tensile stress–strain curves for both adhesives are shown in Figure 14.

**Figure 15:**
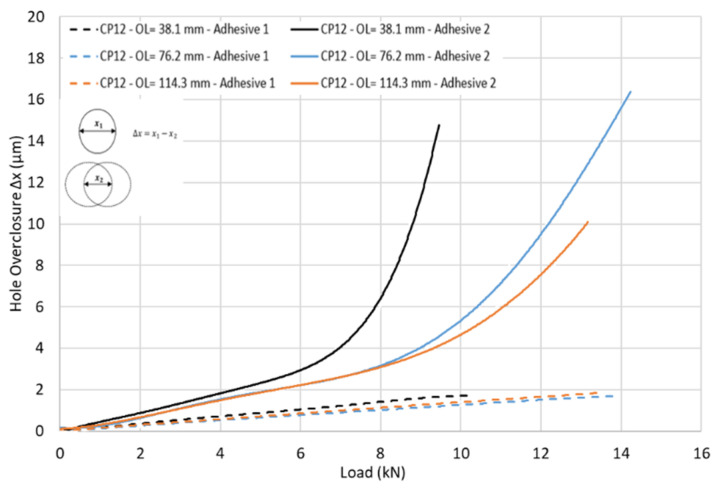
Hole overclosure for various overlap lengths and adhesives in CP12-HBB joints.

Figure 15 shows the hole overclosure for three different overlap lengths (OL) of 38.1 mm, 76.2 mm, and 114.3 mm, all using CP12-HBB joints with an e/d ratio of 3. The bolts were removed from the models to see how the top and bottom laminate holes close up as the tensile load is applied to the laminates. It should be noted that due to a shorter OL of 38.1 mm, only one hole was considered for the model, while for the longer OL of 76.2 and 114.3 mm, two holes were included. An overclosure of 0 would mean that the holes from the top and bottom laminates are perfectly aligned, and as such, the clearance between the bolt shank and the laminate is equivalent to the initial bolt–hole clearance. To achieve load-sharing between the bolts and the adhesive, the hole overclosure must be at least the value of the initial bolt–hole clearance.

**Figure 16:**
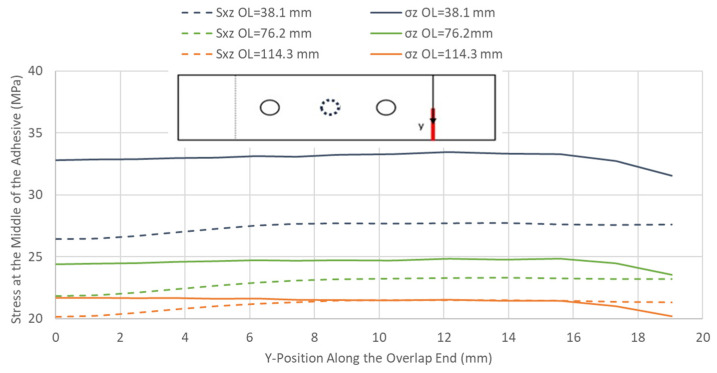
Peel σ_z_ and shear S_xz_ stresses at the overlap end along the adhesive edge for various overlap lengths in CP12-HBB joints using adhesive 1 at 10 kN.

For adhesive 1, a brittle adhesive, very slight hole overclosure is achieved before joint failure. Therefore, this adhesive, independently of the overlap length, shows no promise of potential load sharing between the bolts and the adhesive. On the other hand, adhesive 2, which displays a high plastic deformation, has more hole overclosure, especially at medium to high load levels. It can be noted that adhesive 2 has a Young’s Modulus around four times lower than adhesive 1. Initially, even in the absence of plastic deformation, the hole overclosure for the stiffer adhesive is lower than for adhesive 2, since the lower adhesive stiffness permits greater shear deformations of the adhesive layer, increasing the relative displacement between the top and bottom laminates. For shorter overlap lengths, the hole overclosure is higher at the same load, since a significant hole overclosure occurs only when plastic deformation in the adhesive, which starts at the overlap ends, reaches the hole. For that reason, shorter overlap lengths benefit from increased hole overclosure at lower load levels, as the shorter overlap length increases, especially the peel stresses in the adhesive layer, resulting in premature plastic deformation, as shown in Figure 16. 

It is shown in Table 4 that as the overlap length increases, the joint’s design failure load increases due to the lower stresses in the adhesive layer. However, increasing the overlap length is more beneficial in reducing the peel stresses, as the difference between the shear and peel stresses is more significant for shorter overlap lengths. Still, it can be seen in Figure 16 that increasing the overlap length becomes less efficient at reducing the shear stresses in the adhesive layer.

**Figure 17:**
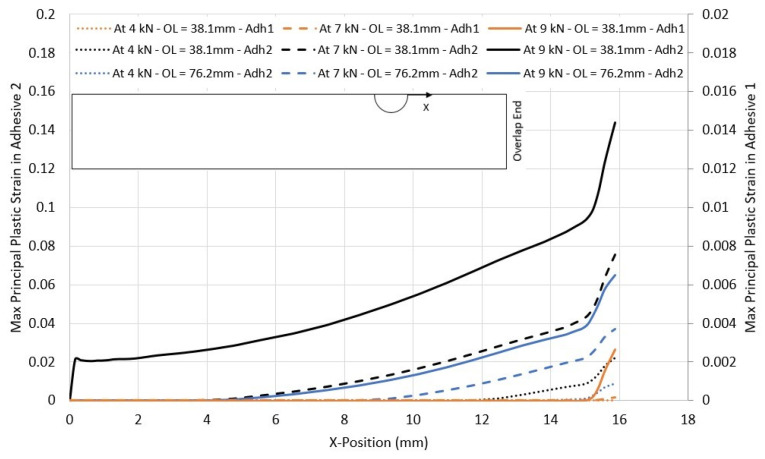
Maximum principal plastic strain for different overlap lengths and adhesives in CP12-HBB joints.

As mentioned earlier, plastic deformation plays a crucial role in the level of hole overclosure displayed by the joint. To highlight this, the maximum principal plastic strain was monitored from the hole edge to the overlap end, as shown in Figure 17. The plastic strain develops initially at the overlap ends, as they are the most stressed regions in the adhesive. Moreover, when the applied load increases, the plastic strains in the adhesive increase. However, this increase and even the apparition of plasticity depend highly on the overlap length and the adhesive used. As the overlap length increases, the stresses developing in the adhesive are reduced, and as such, the development of plastic behaviour is delayed. It can be seen that very little plasticity is developed in adhesive 1, which was qualified earlier as brittle, even at higher load levels (note the scale 10 times smaller compared to adhesive 2).

**Figure 18:**
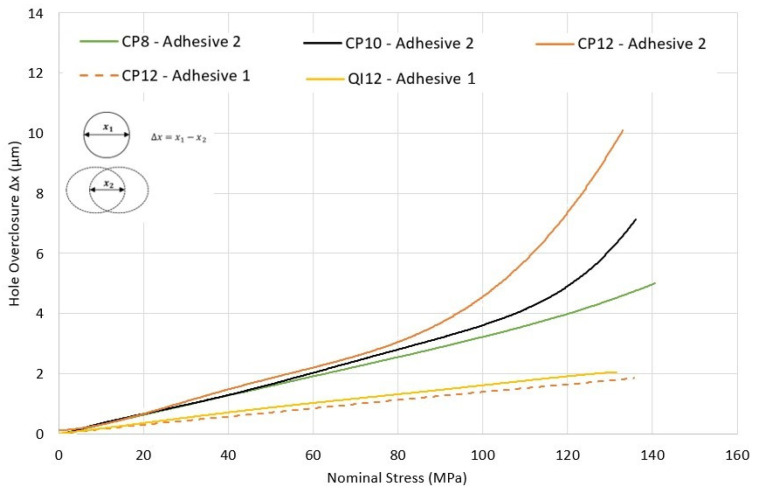
Hole overclosure for different joint parameters and adhesives in HBB with an OL of 114.3 mm.

Laminate thickness and layup type, as two additional parameters impacting load sharing, were monitored in Figure 18. The thinner laminates produced a slightly lower-hole overclosure of the joint. This is explained by the lower stresses developing in the adhesive resulting from the lower secondary bending generated by the reduction in the eccentricity in the load path. As such, in the event of a load-sharing occurrence, the thicker laminate would perform better than a thinner one. Additionally, a QI layup produced greater hole overclosure than a CP layup using the same adhesive and configuration. This is because QI layups generate more stress in the adhesive layer than CP layups, which increases the plastic deformation, resulting in greater hole overclosure.

For the studied parameters, decreasing the adhesive yield strength and modulus with highly plastically deformable properties and using QI layups instead of CP layups increased the load-sharing potential of the joint. While other parameters did increase the hole overclosure, such as the decrease in the overlap length and the increase in the laminate thickness, they were considered unproductive. This is because, although greater hole overclosure is achieved at lower load levels, joint failure is decreased, as these parameters rely on increasing the stress levels in the adhesive layer.

### 3.3. Influence of e/d

**Figure 19:**
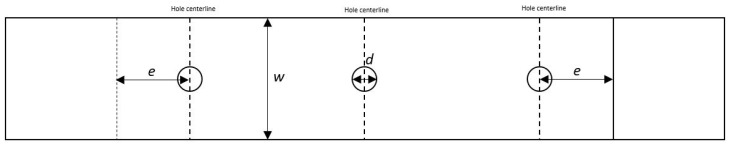
Geometric parameters.

**Figure 20:**
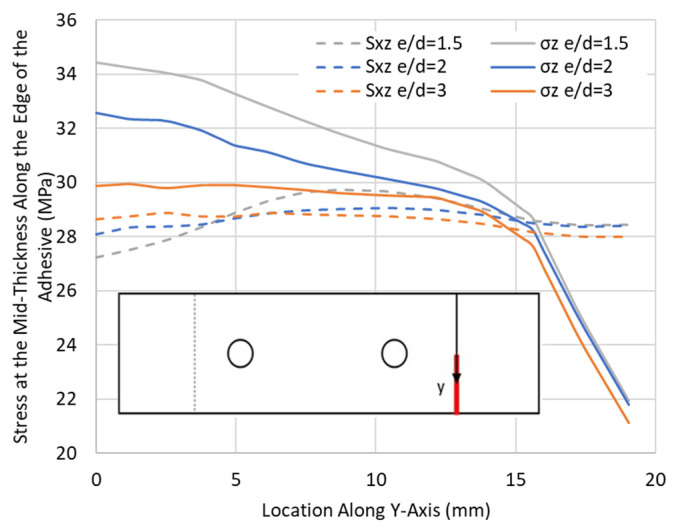
Peel σ_z_ and shear S_xz_ stresses at the overlap end along the adhesive edge in QI12-HBB joints with various e/d ratios at 12.5 kN.

Figure 19 depicts parameters including the edge margin (e) and bolt hole diameter (d). A study on the effect of the variation in the e/d ratio, while neglecting the middle bolt in the HBB configuration, was conducted to see whether placing the bolts closer to the overlap ends would delay adhesive failure. As shown in Table 5, a reduction in the e/d ratio resulted in a lower failure load of the joint for CP and QI layups. Furthermore, not only was the failure stress reduced with a decrease in the e/d ratio, but also, in the case of the CP layup, it changed the failure mode from fibre failure to adhesive failure. Figure 20 shows the peel and shear stresses, monitored at the overlap ends, slightly before failure in the QI12-HBB joints. While the shear stresses in the adhesive are not affected considerably by the e/d ratio, it is noted that a lower e/d ratio results in a significant increase in the peel stress.

**Figure 21:**
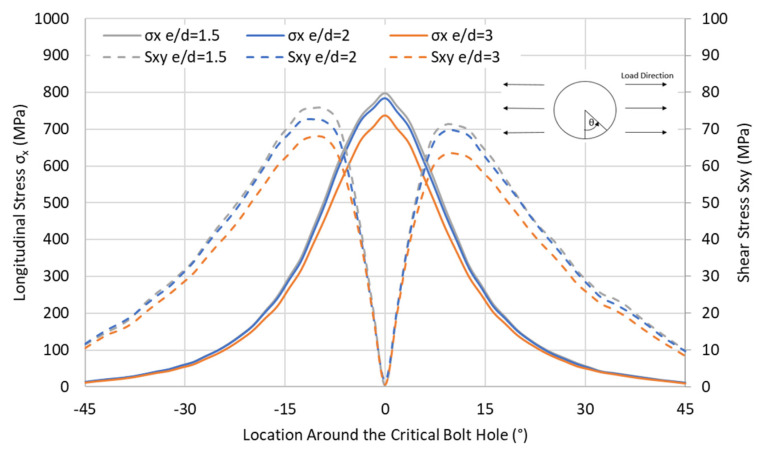
Tensile σx and shear stress Sxy distribution with various e/d ratios around the critical hole in QI12-HBB joints at 12.5 kN.

Not only are the peel stresses increased in the adhesive for shorter e/d ratios, but the longitudinal and shear stresses at the hole are also significantly increased, as shown in Figure 21, for the QI case. It appears that for an e/d ratio of less than 3, the stress concentrations, due to overlap end proximity, become more significant, and this increase is mainly apparent from a change in the e/d between 3 and 2. Therefore, not only does reducing the edge distance result in more peel stresses in the adhesive, but it also significantly increases the stress concentrations at the hole.

### 3.4. Influence of Washer Size

**Figure 22:**
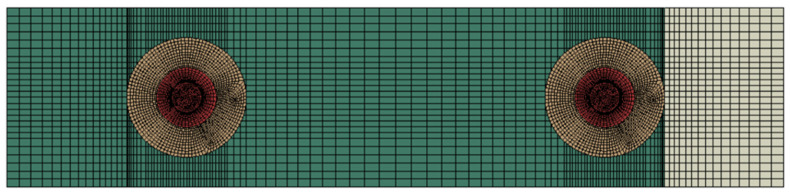
Mesh plot for large washer configuration for QI12-HBB (e/d = 2).

To study the effect of the washer size on the stresses in the adhesive layer, a configuration was analysed for a QI12-HBB joint with an e/d = 2 considering two bolts and a washer with a diameter equivalent to twice the edge distance for each bolt as shown in Figure 22, such that the overlap end is partly covered. The results were then compared with the same configuration, considering the initial washer size. 

**Figure 23:**
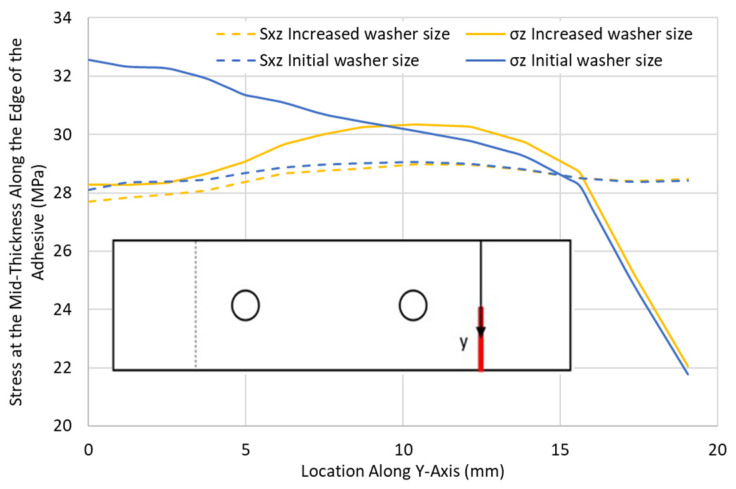
Peel σ_z_ and shear S_xz_ stresses at the overlap end along the adhesive edge in QI12-HBB joints with different washer sizes at 12.5 kN.

Figure 23 demonstrates that the increase in the washer size did not affect the shear stress distribution in the adhesive layer. However, a peel stress reduction of around 13% was noted for the region directly under the washer. As the washer curved away from the overlap end, its effect on the peel stress reduced significantly, to the point where no difference could be noted between the values of the peel stresses in the two joints using different washer sizes. This highlights the fact that the clamping effect of the bolts is limited to the area under the washer. Therefore, to reduce the peel stress in the adhesive using bolts, the clamped area must be directed at the overlap ends, or no peel stress reduction will be observed. It is important to highlight that the progression of damage can be significantly influenced by the appropriate design of washers. Well-designed washers can slow crack propagation compared to a bonded joint, where cracks can freely progress in any direction without any obstacles.

## 4. Discussion and Conclusions

An examination of the mechanics of hybrid bolted/bonded joints based on finite element analysis was performed to understand their behaviour at critical locations around the holes and through the adhesive edges. It was identified that the stresses were concentrated at the hole closer to the grip side in the laminates and at the overlap ends in the adhesive. Cross-ply (CP) layups benefit more from hybridisation than quasi-isotropic (QI) layups due to their higher notch sensitivity. For longer overlap lengths, typically requiring more than two bolts, it was identified that the middle bolts did not contribute to increasing the strength of the HBB joints since the critical regions are at the overlap ends. The QI layups were identified as having greater peel and shear stresses in the adhesive than the CP layups for the same load level due to their lower stiffness, leading to greater out-of-plane displacement and a lower HBB joint strength. A demonstration of the effect of the choice of adhesive on the load-sharing potential of an HBB joint was conducted, highlighting the fact that a low-modulus highly plastic deformable adhesive was required to achieve significant load sharing, as a high level of plastic deformation must occur to have considerable hole overclosure. The overlap length also proved to affect the load-sharing potential of the HBB joints, as longer overlap lengths delay adhesive plastic behaviour, which in turn delays the moment of significant hole overclosure. The increase in laminate thickness also contributes to an increase in the hole overclosure caused by the increase in load eccentricity, leading to higher stresses developed in the adhesive layer that trigger plasticity. Moreover, QI layups appeared to be more promising at achieving load sharing compared to CP layups, as they induce greater stress in the adhesive layer, leading to higher hole overclosure. 

The investigation of HBB joints continued to identify the parameters for optimising their performance. Specifically, the study focused on the effect of the e/d ratio and the size of the washer. It was noted that locating the bolts closer to the overlap ends led to increased peel stresses in the adhesive layer. Moreover, a decrease in the e/d ratio produced a significant increase in the stress concentrations at the critical hole. Through varying the washer size, it was found that to have a significant peel stress reduction, the washer should be in close proximity to the overlap ends. This confirms that the effect of the bolt preload is limited to the region under the washer. 

It would be interesting to perform a finite element analysis studying the joint behaviour in fatigue when the adhesive has been fully plasticized in HBB joints, requiring load-sharing. Under static loading, the plasticization of the adhesive layer helped achieve load-sharing between the bolts and the adhesive. However, during repetitive load cycles, plastic deformation in the adhesive may be detrimental to the joint’s strength and reduce its fatigue life. Therefore, a change in load-sharing initiation may also be noted when the adhesive is initially fully plasticized. 

## Figures and Tables

**Table 1:** Layup configurations [20].

CP Symmetric Sequence-12 Plies (CP12)	QI Symmetric Sequence-12 Plies (QI12)
[(0/90)/(0/90)/(0/90)/(0/90)/(0/90)/(0/90)]_S_	[(0/90)/(±45)/(0/90)/(±45)/(0/90)/(±45)]_S_

**Table 2:** Mesh convergence study at the overlap ends in HBB joints.

Element Size	Applied Load at Damage Initiation (kN)	Difference (%)
0.845	18.93	16.0
0.420	16.33	4.1
0.280	15.68	0.8
0.140	15.56	

**Table 3:** Failure load for different HBB joints with and without middle bolts.

Model	Failure Mode	Load at Failure (KN)
3-Bolts e/d = 3 (CP12)	Fibre Failure	15.41
2-Bolts e/d = 3 (CP12)	Fibre Failure	15.30
3-Bolts e/d = 3 (QI12)	Adhesive Failure	13.78
2-Bolts e/d = 3 (QI12)	Adhesive Failure	13.94

**Table 4:** Failure load of the HBB joint using adhesive 1 for different overlap lengths.

Overlap Length (OL)	Failure Load (KN)	
	CP12-HBB	QI12-HBB
38.1	10.42	9.74
76.2	13.79	12.74
114.3	15.41	13.78

**Table 5:** HBB joint failure load for various e/d ratios.

Model	Failure Mode	Load at Failure (kN)
2-Bolts e/d = 3 (CP12)	Fibre Failure	15.30
2-Bolts e/d = 3 (QI12)	Adhesive Failure	13.94
2-Bolts e/d = 2 (CP12)	Fibre Failure	14.86
2-Bolts e/d = 2 (QI12)	Adhesive Failure	13.21
2-Bolts e/d = 1.5 (CP12)	Adhesive Failure	14.24
2-Bolts e/d = 1.5 (QI12)	Adhesive Failure	12.73

## Data Availability

Data is contained within the article.
